# ﻿The complete mitochondrial genomes of five Agrilinae (Coleoptera, Buprestidae) species and phylogenetic implications

**DOI:** 10.3897/zookeys.1092.80993

**Published:** 2022-04-06

**Authors:** Zhonghua Wei

**Affiliations:** 1 The Key Laboratory of Southwest China Wildlife Resources Conservation of the Ministry of Education, College of Life Sciences, China West Normal University, 637009, Nanchong, Sichuan Province, China China West Normal University Nanchong China

**Keywords:** Comparative analysis, mitogenome, phylogenetic analysis

## Abstract

Five complete mitochondrial genomes of five species from the subfamily Agrilinae were sequenced and annotated, including *Coraebusdiminutus* Gebhardt, 1928 (15,499 bp), *Coraebuscloueti* Théry, 1893 (15,514 bp), *Meliboeussinae* Obenberger, 1935 (16,108 bp), *Agrilussichuanus* Jendek, 2011 (16,521 bp), and *Sambusfemoralis* Kerremans, 1892 (15,367 bp). These mitogenomes ranged from 15,367 to 16,521 bp in length and encoded 37 typical mitochondrial genes: 13 protein-coding genes (13 PCGs), 2 ribosomal RNA genes (2 rRNAs), 22 transfer RNA genes (22 tRNAs), and a control region (CR). Most of PCGs had typical ATN start codons and terminated with TAR or an incomplete stop codon T–. Among these five mitogenomes, Leu2, Ile, Phe, Ser2, Gly, Met, and Val were the seven most frequently encoded amino acids. Interestingly, in *A.sichuanus*, a 774 bp insertion was present at *trnW* and *trnC* junction, which is unusual in Buprestidae. Additionally, phylogenetic analyses were performed based on three kinds of nucleotide matrixes (13 PCGs, 2 rRNAs, and 13 PCGs + 2 rRNAs) using Bayesian inference and maximum-likelihood methods. The results showed that the clade of Buprestidae was well separated from outgroups and all Agrilinae species formed to a single highly supported clade. The tribe Coraebini was polyphyletic, as the genus *Meliboeus* (Coraebini) clustered with the genus *Trachys* (Tracheini). The rRNA genes had important impact for the tree topology of Agrilinae. Compared to the tribes Tracheini and Agrilini, the tribe Coraebini is a younger group.

## ﻿Introduction

The superfamily Buprestoidea, which contains the families Buprestidae and Schizopodidae, differs from other groups of the Elateriformia by their serrate antennae, hypognathous head, transverse suture of metaventrite present, and two connate basal abdominal ventrites (Bellamy and Volkovitsh 2016). The buprestid beetles are a large group containing six subfamilies, 521 genera, and more than 15,000 species widely distributed in the world (Bellamy 2008; Kubáň et al. 2016). The adults exhibit a broad range of host utilization in leaves, flowers, and stems, whereas larvae are mostly internal feeders on roots and stems, or feed on foliage of woody or herbaceous plants (Bellamy and Volkovitsh 2016). Only adults of the Australian *Xyrosceliscrocata* were reported to feed on the sap of the host plant *Macrozamiacommunis* (Bellamy 1997).

Although taxonomists have made important contributions to the buprestid classification of subfamilies and tribes based on several morphological characteristics (Cobos 1980, 1986; Tôyama 1987; Hołyński 1988, 1993, 2009; Bellamy 2003), the problems of the overall classification in Buprestoidea remain unsettled.

In the past two decades, molecular systematic approaches have been used to resolve unsettled classification and phylogenetic relationships in Insecta (Short and Fikáček 2013; Cline et al. 2014; Robertson et al. 2015; Kundrata et al. 2017; Gimmel et al. 2019; Lee et al. 2020). As to Buprestidae, Bernhard et al. (2005) first used molecular phylogenetic methods based on three mitochondrial markers (*nad1*, *12S*, and *16S*) and confirmed that the *Agrilusviridis* complex, which is widely distributed across Eurasia, is monophyletic. Pentinsaari et al. (2014) and Pellegrino et al. (2017) used mitochondrial markers to evaluate the diversity of *A.viridis* complex, their results suggest that different feeding forms of *A.viridis* represent distinct species. Subsequently, Evans et al. (2015) performed the first large-scale phylogenetic trees combing nuclear and mitochondrial data from 141 species to understand the higher-level relationships in Buprestidae. In that study, the monophyly of the family Schizopodidae and subfamilies Agrilinae, Julodinae, and Galbellinae were strongly supported, while the interrelationships of Chrysochroinae and Buprestinae remained uncertain. Hansen et al. (2016) used molecular systematic methods based on nuclear and mitochondrial data (*coi* and *ak*) to investigate the relationships within *Chrysobothrisfemorata* species group, and their results showed that some morphological species were not well separated. Kelnarova et al. (2019) provided a molecular phylogeny of *Agrilus* species from the Northern Hemisphere and their results suggest that DNA barcoding is a powerful species identification to *Agrilus*.

During this time, the mitogenome emerged as a valuable source for higher-level phylogenetic analyses, evolutionary strategies, and genetic diversity analyses (Saccone et al. 1999; Krzywinski et al. 2011; Cameron 2014; Qin et al. 2015; Song et al. 2019; Wang et al. 2019). Several buprestid mitogenomes have been sequenced and reported, such as the mitogenome of *Chrysochroafulgidissima* (Schönherr, 1817) by Hong et al. (2009); the mitogenome of *Agrilusplanipennis* Fairmaire, 1888 by Duan et al. (2017), who also performed phylogenetic analyses based on 13 PCGs of 45 mitogenomes of coleopterans; the mitogenome of *Trachysvariolaris* Saunders, 1873 by Cao and Wang (2019a); and the mitogenome of *Coraebuscavifrons* Descarpentries & Villiers, 1967 by Cao and Wang (2019b). More detailed information of buprestid mitogenomes is presented in Table 1.

**Table 1.** Information on the mitogenomes of Buprestidae and two outgroups used in this study.

No.	Taxa	Accession no.	Genome size (bp)	A%	A+T%	AT skew	GC skew	References
1	*Coraebusdiminutus*	OK189521	15,499	38.34	68.42	0.12	−0.25	This study
2	*Coraebuscloueti*	OK189520	15,514	38.53	69.27	0.11	−0.25	This study
3	*Meliboeussinae*	OK189522	16,108	40.18	72.42	0.11	−0.22	This study
4	*Sambusfemoralis*	OK349489	15,367	40.98	73.23	0.12	−0.18	This study
5	*Agrilussichuanus*	OK189519	16,521	40.19	71.73	0.12	−0.21	This study
6	*Agrilusplanipennis*	KT363854	15,942	40.25	71.90	0.12	−0.24	Duan et al. 2017
7	*Agrilusmali*	MN894890	16,204	40.34	74.46	0.08	−0.18	Sun et al. 2020
8	*Coraebuscavifrons*	MK913589	15,686	38.94	69.79	0.12	−0.18	Cao and Wang 2019b
9	*Trachysauricollis*	MH638286	16,429	38.94	71.05	0.10	−0.20	Xiao et al. 2019
10	*Trachystroglodytiformis*	KX087357	16,316	41.03	74.62	0.10	−0.19	Unpublished
11	*Trachysvariolaris*	MN178497	16,771	39.92	72.11	0.11	−0.21	Cao and Wang 2019a
12	*Melanophilaacuminata*	MW287594	15,853	38.74	75.66	0.02	−0.25	Peng et al. 2021
13	*Anthaxiachinensis*	MW929326	15,881	40.12	73.61	0.09	−0.29	Chen et al. 2021
14	*Chrysochroafulgidissima*	EU826485	15,592	40.31	69.92	0.15	−0.24	Hong et al. 2009
15	*Acmaeodera* sp.	FJ613420	16,217	38.11	68.41	0.11	−0.25	Sheffield et al. 2009
16	*Heterocerusparallelus* (outgroup)	KX087297	15,845	41.90	74.03	0.13	−0.24	Unpublished
17	*Dryopsernesti* (outgroup)	KX035147	15,672	39.04	72.98	0.07	−0.23	Unpublished

Currently, the subfamily Agrilinae contains four tribes (Agrilini, Coraebini, Aphanisticini, and Tracheini); however, the phylogenetic placement of several genera of this subfamily remains unstable. The genera in the tribes Coraebini and Agrilini were revised by Kubáň et al. (2000). In that study, the genus *Sambus* in the tribe Coraebini was transferred to Agrilini based on the female behavior of ovipositing on rather smooth surfaces of living plants. Later, Kubáň (2016) placed the genera *Sambus*, *Parasambus*, and *Pseudagrilus* in *incertae sedis*. In order to solve these problems, we contribute mitogenomic data of five species of buprestids, *Coraebusdiminutus* Gebhardt, 1928, *Coraebuscloueti* Théry, 1893, *Meliboeussinae* Obenberger, 1935, *Agrilussichuanus* Jendek, 2011, and *Sambusfemoralis* Kerremans, 1892, and perform a molecular phylogenetic analysis in this study. The phylogenetic trees of 15 species from nine genera belonging to four subfamilies of Buprestidae were constructed based on the newly sequenced and previously reported mitogenomes (Table 1).

## ﻿Material and methods

### ﻿Sampling and DNA extraction

Specimens of five species were collected using an entomological net. Among them, *C.diminutus*, *C.cloueti*, *M.sinae*, and *A.sichuanus* were collected in the Dayaoshan Mountains in Guangxi Zhuang Autonomous Region, and *S.femoralis* was collected at Yingjiang County in Yunnan Province, China. Specimens were immediately preserved in 95% ethanol in the field after collected and then stored at –24 °C in the laboratory. The specimens were identified based on morphological characteristics under a Leica M205 FA stereomicroscope. Total DNA was extracted from muscle tissues using the Ezup Column Animal Genomic DNA Purification Kit (Shanghai, China) following the manufacturer’s instructions.

### ﻿Sequencing, sequence assembly, annotation, and heterogeneity

DNA sequencing and de novo assembly of each mitogenome were performed by Beijing Aoweisen Gene Technology Co. Ltd (Beijing, China). 22 tRNA genes were identified using the MITOS webserver, with the parameters of the Invertebrate Mito genetic code (Bernt et al. 2013). Their secondary structures were plotted manually from the MITOS predictions using Adobe Illustrator. Every sequence of tRNA genes was manually checked separately. The PCGs were identified as open reading frames corresponding to the 13 PCGs. The rRNAs and control regions were identified by the boundaries of the tRNA genes. The tRNA secondary structures were identified using tRNAscan-SE (Lowe and Chan 2016). Mitogenome maps (Suppl. material 1: Fig. S1) were produced using Organellar Genome DRAW (OGDRAW) (Greiner et al. 2019). The Base composition and relative synonymous codon usage values were determined using MEGA 6.0 (Kumar et al.2016). Strand asymmetry was calculated using the formulae AT-skew = (A – T) / (A + T), and GC-skew = (G – C) / (G + C) (Perna and Kocher 1995). In the control region (CR), tandem repeat elements were detected by Tandem Repeats Finder (Benson 1999). The heterogeneous analysis of the 13 PCGs and two rRNAs datasets were performed using AliGROOVE 1.06 (Kück et al. 2014), and the nucleotide diversity (Pi) and the ratio of Ka/Ks of PCGS were calculated with DnaSP v. 5 (Librado and Rozas 2009).

### ﻿Phylogenetic analyses

Phylogenetic trees for *A.sichuanus*, *C.diminutus*, *C.cloueti*, *M.sinae*, *S.femoralis*, and 10 other buprestid species belonging to four subfamilies were reconstructed by three separate datasets (13 PCGs, 2 rRNAs, and 13 PCGs + 2 rRNAs) using different best-fit models (Table 4). The mitogenomes of *Heterocerusparallelus* (Heteroceridae) and *Dryopsernesti* (Dryopidae) were used as outgroups, as they are phylogenetically distant from Buprestidae in the suborder Polyphaga (Xiao et al. 2019). The phylogenetic analyses were performed using PhyloSuite v. 1.2.2 (Zhang et al. 2020). Nucleotide sequences of the 13 PCGs and 2 rRNAs of all 17 mitogenomes were aligned using ClustalW (Thompson et al. 1994) and trimmed using trimAl v. 1.2 (Capella-Gutiérrez et al. 2009). The best-fit model for three datasets was determined by ModelFinder based on Bayesian information criterion. The maximum-likelihood (ML) and Bayesian inference (BI) methods were used to reconstruct the phylogenetic trees by IQ-tree v. 1.6.8 (Guindon et al. 2010) and MrBayes v. 3.2.6 program respectively (Ronquist et al. 2012). Bayesian analyses were run with two independent chains spanning 2,000,000 generations, four Markov chains, sampling at every 100 generations, and a burn-in period of 0.25 for each chain. The phylogenetic trees were edited and visualized by Figtree v. 1.4.3.

## ﻿Results and discussion

### ﻿Genome organization and base composition

The complete mitogenomes of the buprestids *A.sichuanus*, *C.diminutus*, *C.cloueti*, *M.sinae*, and *S.femoralis* have the following GenBank accession numbers attributed to them: OK189519, OK189521, OK189520, OK189522, OK349489. The mitogenomes of these five species contained the 37 typical mitochondrial genes (13 PCGs, 22 tRNAs, and 2 rRNAs) and a control region (CR) (Table 2). The composition and arrangement of the mitochondrial genes in these species (Table 2) were highly similar as those in most other buprestid species (Duan et al. 2017; Cao and Wang 2019a, 2019b; Xiao et al. 2019; Chen et al. 2021; Peng et al. 2021).

**Table 2.** The five newly annotated Buprestidae mitogenomes. The order of these five species in the table is as follows: *Agrilussichuanus*, *Coraebusdiminutus*, *Coraebuscloueti*, *Meliboeussinae*, and *Sambusfemoralis*. – not determined.

Gene	Strand	Position From	To	Start codons	Stop condons	Anticodon	Intergenic nucleotides
*trnI*	J	1/1/1/1/1	65/63/63/64/65			GAT	-3/-3/-3/5-3
*trnQ*	N	63/61/61/70/63	131/129/129/138/131			AAG	-1/0/0/0/-1
*trnM*	J	131/129/129/138/131	199/196/196/205/196			CAA	0/0/0/0/0
*nad2*	J	200/197/197/206/197	1222/1219/1219/1231/1210	ATC/ATT/ATT/ATC/ATT	TAA/TAG/TAA/TAA/TAA		1/1/-2/0/-2
*trnW*	J	1224/1221/1218/1232/1209	1293/1286/1283/1303/1273			ACA	774/-8/-13/13/-8
*trnC*	N	2068/1279/1276/1296/1266	2130/1339/1336/1356/1326			GCA	0/2/2/0/0
*trnY*	N	2131/1342/1339/1357/1327	2195/1404/1401/1419/1387			GAA	9/1/1/1/1
*cox1*	J	2205/1406/1403/1421/1389	3735/2936/2933/2951/2919	–/–/–/–/–	TAA/TAA/TAA/TAA/TAA		0/0/0/0/0
*trnL2*	J	3736/2937/2934/2952/2920	3802/3003/3001/3016/2984			AAG	0/0/0/0/0
*cox2*	J	3803/3004/3002/3017/2985	4484/3670/3668/3698/3666	ATT/ATA/ATA/ATC/ATT	TAA/TAA/TAA/TAA/TAA		0/0/0/0/0
*trnK*	J	4485/3671/3669/3699/3667	4553/3740/3738/3768/3736			CAA	0/0/0/0/0
*trnD*	J	4554/3741/3739/3769/3737	4618/3803/3802/3830/3798			GAC	0/0/0/0/0
*atp8*	J	4619/3804/3803/3831/3799	4777/3962/3961/3989/3954	ATT/ATA/ATC/ATT/ATA	TAG/TAA/TAA/TAA/TAG		0/-7/-7/-7-7
*atp6*	J	4771/3956/3955/3983/3948	5445/4630/4629/4657/4622	ATG/ATG/ATG/ATG/ATG	TAA/TAA/TAA/TAA/TAA		-1/-1/-1/-1/-1
*cox3*	J	5445/4630/4629/4657/4622	6233/5416/5415/5443/5405	ATG/ATG/ATG/ATG/ATG	TAG/TAA/TAA/TAA/TAA		8/0/0/0/0
*trnG*	J	6242/5417/5416/5444/5406	6306/5477/5476/5509/5469			ACC	0/0/0/0/0
*nad3*	J	6307/5478/5477/5510/5470	6660/5831/5830/5863/5823	ATT/ATT/ATT/ATT/ATT	TAG/TAG/TAG/TAG/TAG		-2/-2/-2/-2/-2
*trnA*	J	6659/5830/5829/5862/5822	6721/5890/5889/5924/5884			AGC	0/-1/-1/-1/0
*trnR*	J	6722/5890/5889/5924/5885	6781/5952/5951/5988/5947			ACG	1/-1/-1/-1/1
*trnN*	J	6783/5952/5951/5988/5949	6849/6017/6016/6051/6013			GAA	0/0/0/0/0
*trnS1*	J	6850/6018/6017/6052/6014	6916/6075/6074/6117/6080			ACA	1/0/7/-1/0
*trnE*	J	6918/6076/6082/6117/6081	6982/6139/6143/6179/6143			AAC	-1/-4/-4/-1/-1
*trnF*	N	6982/6136/6140/6179/6143	7045/6198/6202/6240/6207			GAA	0/0/0/0/0
*nad5*	N	7046/6199/6203/6241/6208	8768/7915/7919/7960/7915	ATA/ATT/ATT/ATT/ATA	TAA/TAA/TAA/TAA/TAA		0/0/0/0/0
*trnH*	N	8769/7916/7920/7961/7916	8830/7977/7981/8026/7978			GAG	0/0/0/0/0
*nad4*	N	8831/7978/7982/8027/7979	10,166/9295/9299/9362/9308	ATG/ATG/ATG/ATG/ATG	TAA/TAA/TAA/TAA/TAA		-7/-7/-7/-7/-7
*nad4L*	N	10,160/9289/9293/9356/9302	10,444/9576/9580/9640/9589	ATG/ATG/ATG/ATG/ATA	TAA/TAA/TAA/TAA/TAA		4/3/3/2/1
*trnT*	J	10,449/9580/9584/9643/9591	10,511/9642/9646/9704/9654			AGA	-1/-1/-1/-1/-1
*trnP*	N	10,511/9642/9646/9704/9654	10,574/9704/9708/9769/9717			AGG	1/1/1/1/1
*nad6*	J	10,576/9706/9710/9771/9719	11,079/10,185/10,189/10,259/10,192	ATT/ATA/ATA/ATG/ATT	TAA/TAA/TAA/TAA/TAA		-1/-1/-1/-1/-1
*cytb*	J	11,079/10,185/10,189/10,259/10,192	12,224/11,327/11,331/11,401/11,334	ATG/ATG/ATG/ATG/ATG	TAA/TAG/TAG/TAG/TAG		8/-2/-2/-2/-2
*trnS2*	J	12,233/11,326/11,330/11,400/11,333	12,298/11,391/11,395/11,465/11,400			ACA	17/9/9/19/14
*nad1*	N	12,316/11,411/11,415/11,485/11,415	13,266/12,361/12,365/12,432/12,365	TTG/TTG/TTG/TTG/TTG	TAA/TAA/TAA/TAG/TAA		1/1/1/0/1
*trnL1*	N	13,268/12,363/12,367/12,433/12,367	13,334/12,427/12,431/12,495/12,434			AAG	0/0/0/0/0
*rrnL*	N	13,335/12,428/12,432/12,496/12,435	14,605/13,693/13,697/13,757/13,692				0/0/0/0/0
*trnV*	N	14,606/13,694/13,698/13,758/13,693	14,674/13,762/13,766/13,826/13,761			AAC	0/0/0/0/0
*rrnS*	N	14,675/13,763/13,767/13,827/13,762	15,379/14,480/14,483/14,531/14,457				0/0/0/0/0
A + T rich region		15,380/14,481/14,484/14,532/14,458	16,521/15,499/15,514/16,108/15,367				0/0/0/0/0

Four of the 13 PCGs (*nad1*, *nad4L*, *nad4*, and *nad5*), eight tRNAs (*trnQ*, *trnV*, *trnL1*, *trnP*, *trnH*, *trnF*, *trnY*, and *trnC*), and two rRNAS (*rrnL* and *rrnS*) are encoded on the N-strand, whereas the other 23 genes (9 PCGs and 14 tRNAs) are encoded on the J-strand. The mitogenome sequence of these five buprestid species ranged in size from 15,367 to 16,521 bp.

The mean A + T nucleotide contents of five complete mitogenomes were similar: 68.42% in *C.diminutus*, 69.27% in *C.cloueti*, 72.42% in *M.sinae*, 71.73% in *A.sichuanus*, and 73.23% in *S.femoralis*. The entire mitogenomes had a higher A + T contents of 68.42–73.23% (66.05–72.50% for PCGs, 70.95–74.03% for tRNA genes, 75.20–77.33% for rRNA genes, and 74.17–78.38% for the CR) than G + C contents, which is consistent with the typical base of buprestid mitogenomes. The overall AT skews in these five complete mitogenomes were 0.12, 0.11, 0.11, 0.12, and 0.12, respectively. These five species showed a positive TA skew, suggesting that a slight AT bias which are similar to those observed in other buprestid species (Duan et al. 2017; Cao and Wang 2019a, 2019b; Xiao et al. 2019; Chen et al. 2021; Peng et al. 2021).

### ﻿Protein-coding regions, codon usage, and nucleotide diversity

**Figure 1.**
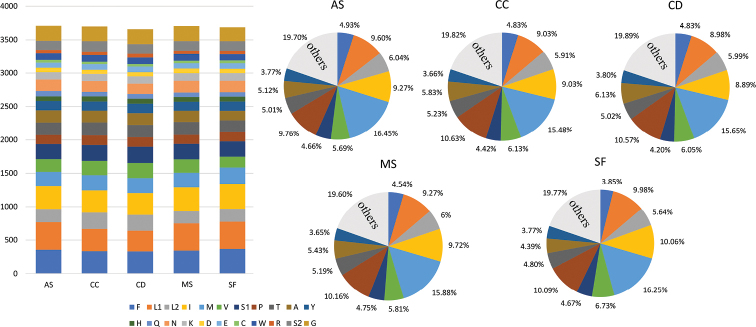
Numbers of different amino acids in the mitogenomes of the five buprestid species; the stop codon is not included. AS: *Agrilussichuanus*, CC: *Coraebuscloueti*, CD: *Coraebusdiminutus*, MS: *Meliboeussinae*, and SF: *Sambusfemoralis*.

**Figure 2.**
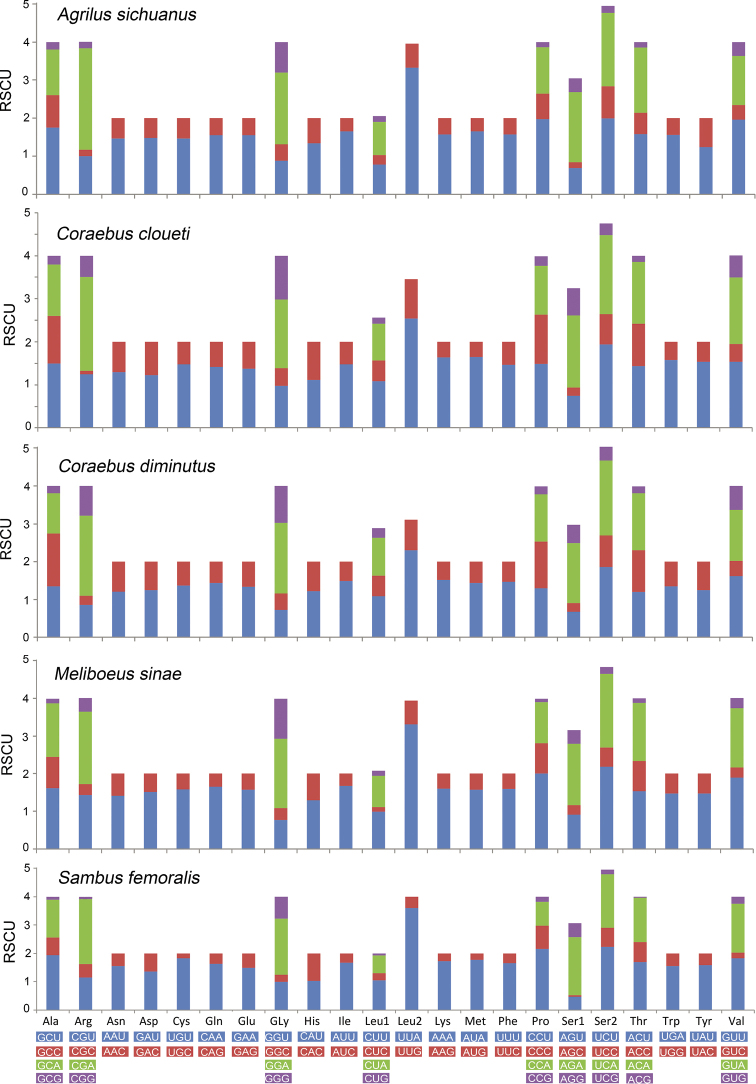
RSCU (relative synonymous codon usage) of the mitogenomes of the five buprestid species; the stop codons are not included.

The total lengths of PCGs in these five buprestid species ranged from 11,090 to 11,158 bp, accounting for 67.54–72.17% of the entire mitogenomes. Similar to the other buprestid mitogenomes, *nad5* and *atp8* were found to be the largest (1708–1723 bp) and smallest (156–159 bp) genes, respectively. The majority of PCGs strictly started with an ATN (ATA/ATT/ATC/ATG) start codon, except for the *nad1* starting with TTG. All PCGs strictly terminated with TAR (TAG/TAA) or an incomplete stop codon T–. Similar to most previously sequenced members of Buprestidae, the AT skew (0.11–0.12) of these five PCGs (Table 3) were similar among the 15 buprestid species. Summaries of the numbers of amino acids in the annotated PCGs and relative synonymous codon usage are presented in Figs 1 and 2. Overall codon usage among the sequenced buprestid mitogenomes was found to be similar, with Leu2, Ile, Phe, Ser2, Gly, Met, and Val being the seven most frequently coded amino acids.

**Table 3.** Summarized mitogenomic characteristics of the five buprestid species in this study.

Species	PCGs			rRNAs			tRNA			CR		
	Size (bp)	A+T content	AT skew	Size (bp)	A+T content	AT skew	Size (bp)	A+T content	AT skew	Size (bp)	A+T content	AT skew
*A.sichuanus*	11,158	70.08	−0.15	1976	75.96	−0.13	1444	74.03	−0.0009	1142	74.17	0.06
*C.diminutus*	11,093	66.05	−0.14	1984	75.20	−0.11	1477	70.95	0.03	1019	77.72	0.02
*C.cloueti*	11,093	67.09	−0.15	1983	75.39	−0.11	1414	71.22	0.019	1031	78.27	0.02
*M.sinae*	11,135	70.70	−0.15	1967	77.33	−0.11	1435	72.13	0.007	1577	78.38	0.13
*S.femoralis*	11,090	72.50	−0.16	1954	75.69	−0.13	1430	73.85	0.03	910	75.82	0.18

**Figure 3.**
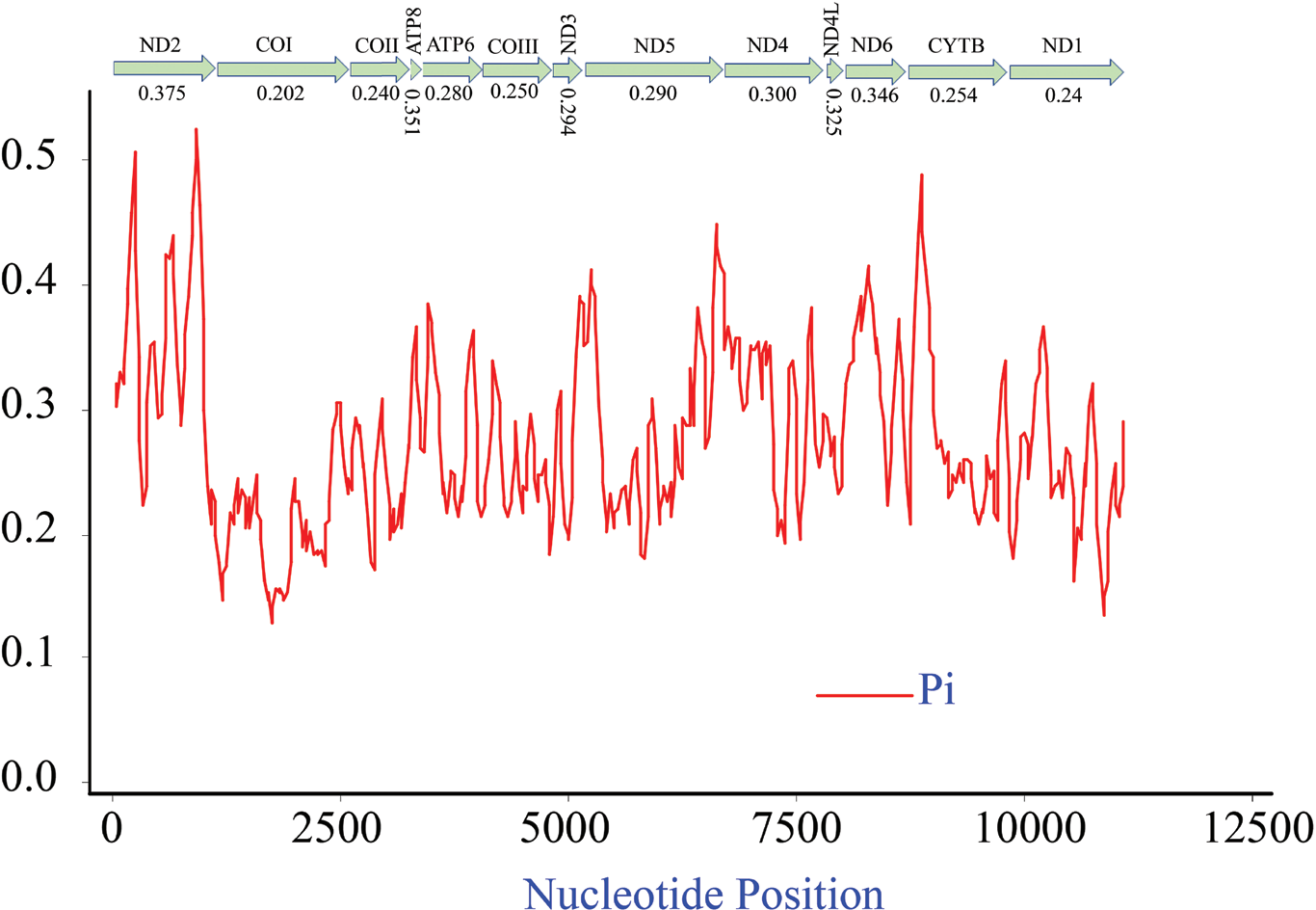
Nucleotide diversity (Pi) of 13 PCGs among five newly sequenced Agrilinae mitogenomes.

**Figure 4.**
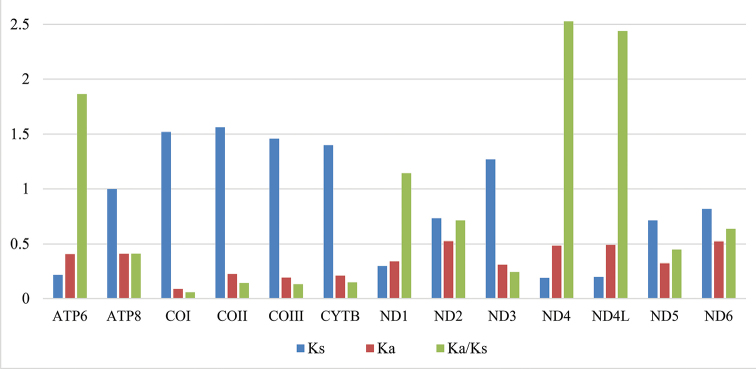
The ratio of Ka/Ks of 13 PCGs among the 15 reported Buprestidae mitogenomes.

The nucleotide diversity (Pi) of the 13 PCGs among five species of Agrilinae is provided (Fig. 3), which ranged from 0.202 to 0.375. In these genes, *nad2* (Pi = 0.375) presented the highest variability, followed by *nad6* (Pi = 0.346), *nad4* (Pi = 0.300), and *nad5* (Pi = 0.290); *cox1* (Pi = 0.20) exhibited the lowest variability. The ratio of Ka/Ks (Fig. 4) for each gene of the 13 PCGs was calculated. The values of *nad4* and *nad4L* are distinctly higher than others, which suggests that the genes *nad4* and *nad4L* have a relatively higher evolutionary rate.

### ﻿tRNA, rRNA genes, and heterogeneity

**Figure 5.**
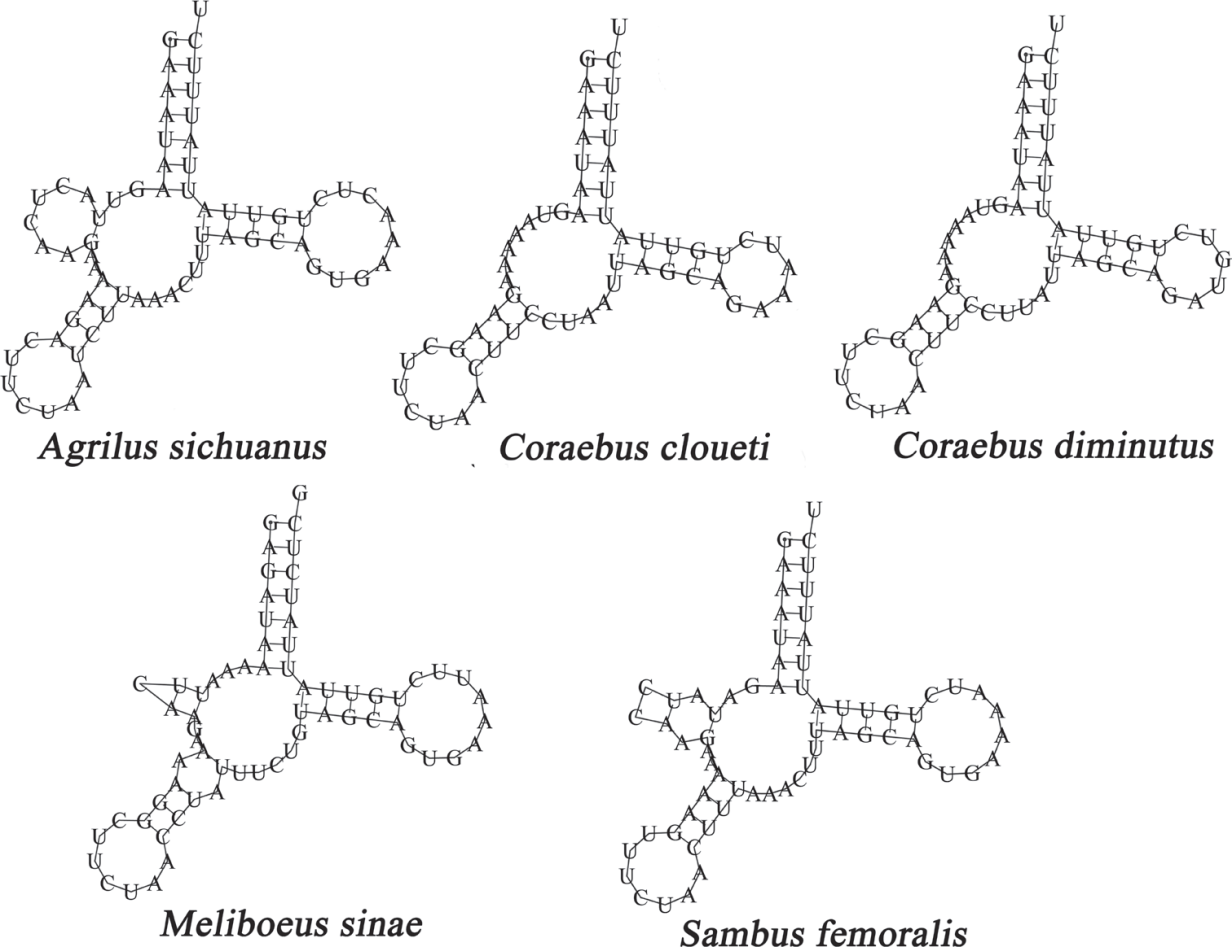
The predicted secondary structures of the tRNA-Ser in the mitogenomes of the five buprestid species.

The length of *rrnL* genes ranged from 1258 bp (*S.femoralis*) to 1271 bp (*A.sichuanus*), whereas *rrnS* ranged from 696 bp (*S.femoralis*) to 718 bp (*C.diminutus*). The A + T content of the rRNA genes ranged from 75.20% (*C.diminutus*) to 77.33% (*M.sinae*) (Table 3). Compared with those in other sequenced buprestid mitogenomes, the rRNA genes in these five newly sequenced buprestid mitogenomes are highly conserved (Hong et al. 2009; Duan et al. 2017; Cao and Wang 2019a, 2019b; Xiao et al. 2019; Sun et al. 2020; Chen et al. 2021; Peng et al. 2021). These rRNAs were located between the CR and *trnL1*, and separated by *trnV*. The total lengths of the 22 tRNA genes ranged from 1414 bp (*C.cloueti*) to 1444 bp (*C.diminutus*), whereas individual tRNA genes typically ranged in size from 58 to 70 bp, among which, eight tRNAs were encoded on the N-strand and the remaining 14 encoded on the J-strand. The secondary structures of tRNAs showed a standard clover-leaf structure (Suppl. material 1: Figs S2–S6), except for tRNA-Ser (Fig. 5) which lacks or has an unusual dihydrouridine arm, which forms a loop commonly found in other insects (Xiao et al. 2011; Park et al. 2012; Yu et al. 2016; Yan et al. 2017; Yu and Liang 2018; Li et al. 2019). In *A.sichuanus*, the longest intergenic nucleotide (774 bp) was located between *trnW* and *trnC*, which is an interesting and specific phenomenon in Buprestidae. The degree of heterogeneity of the 13 PCGs dataset was higher than that of the two rRNAs dataset (Suppl. material 1: Fig. S7). Additionally, the heterogeneity in sequence divergences was slightly stronger for *Coraebus* than for other buprestid genera (Suppl. material 1: Fig. S7).

### ﻿Control region

The CR, also known as the A + T-rich region (Wolstenholme 1992), was the largest non-coding region and located between *trnI* and *rrnS*. The length of CR ranged from 910 bp (*S.femoralis*) to 1577 bp (*M.sinae*). The A + T content (74.17–78.38%) of the CR of these five species was found to be higher than that of the whole genome (68.42–73.23%), PCGs (66.05–72.50%), rRNAs (75.20–77.33%), and tRNAs (70.95–73.85%) (Table 3). Moreover, the compositional analysis revealed that the mitogenomes of the five buprestid species had a positive AT skew (0.02–0.18) in the CR. In these five species, only *C.cloueti* and *C.diminutus* had no tandem repeat element detected; however, those of *A.sichuanus* (20 and 40 bp), *M.sinae* (53 bp), and *S.femoralis* (265 bp) had different lengths.

**Table 4.** Best-fit models of three datasets used for phylogeny.

	ML method	BI method
13 PCGs	GTR+F+I+G4	GTR+F+I+G4
2 rRNAs	TVM+F+I+G4	GTR+F+I+G4
13 PCGs +2 rRNAs	GTR+F+I+G4	GTR+F+I+G4

### ﻿Phylogenetic analyses

**Figure 6.**
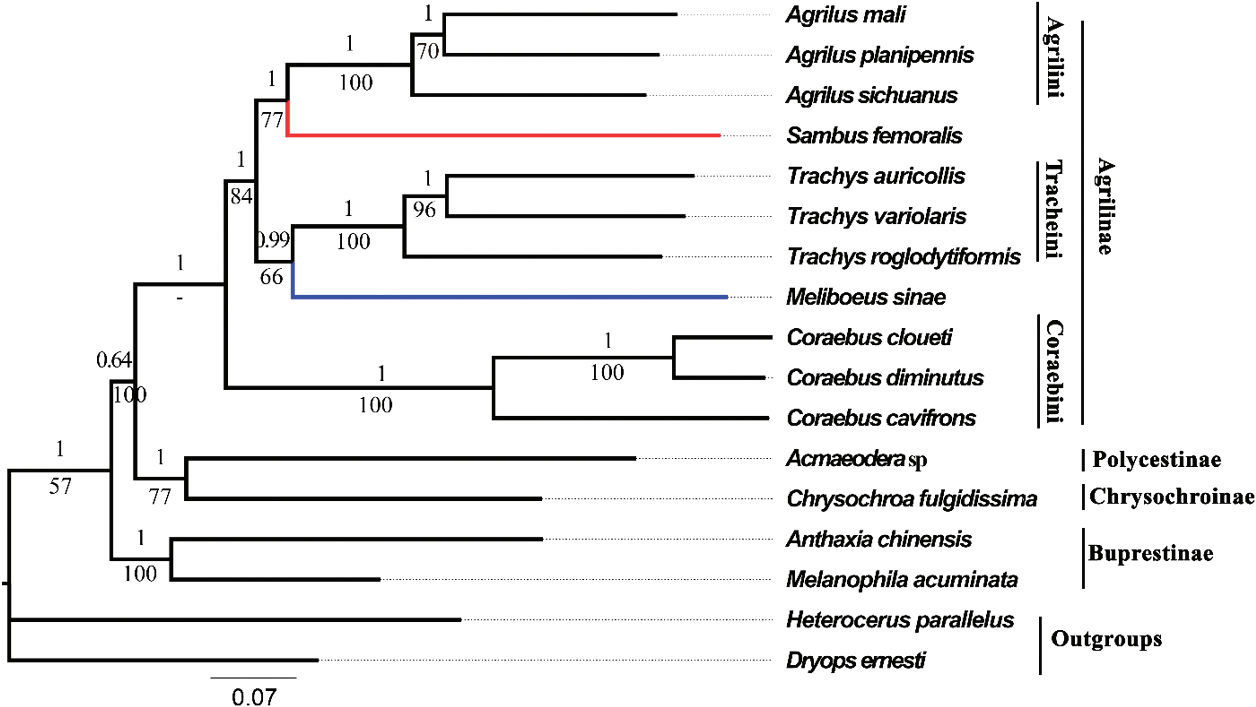
Phylogenetic relationships of 15 selected buprestid species using both BI and ML analyses based on 13 PCGs of mitogenomes. The numbers on the branches show posterior probability (BI tree), whereas the values under branches are bootstrap (ML tree).

**Figure 7.**
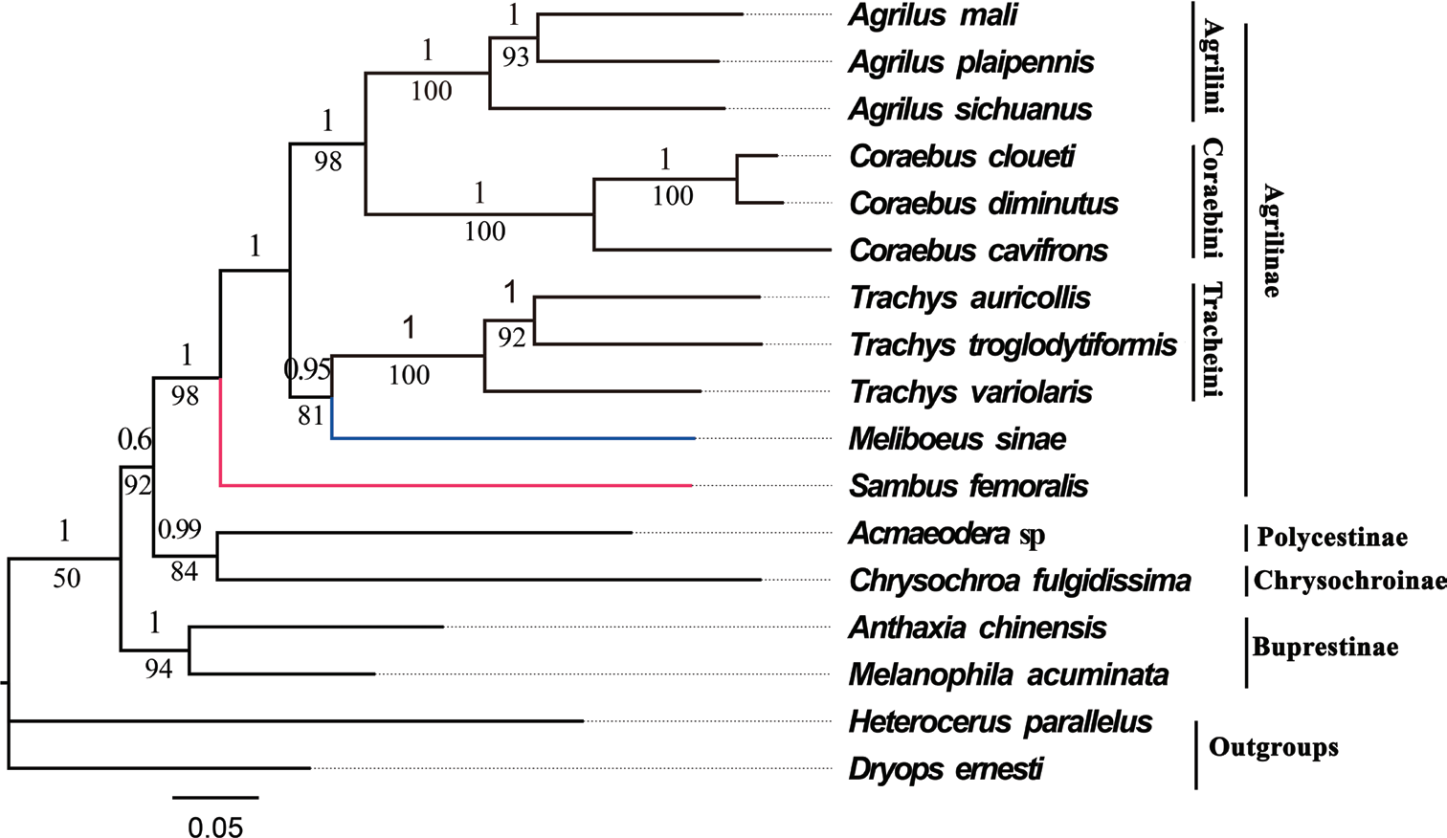
Phylogenetic relationships of 15 selected buprestid species using both BI and ML analyses based on 2 rRNAs of mitogenomes. The numbers on the branches show posterior probability (BI tree), whereas the values under branches are bootstrap (ML tree).

**Figure 8.**
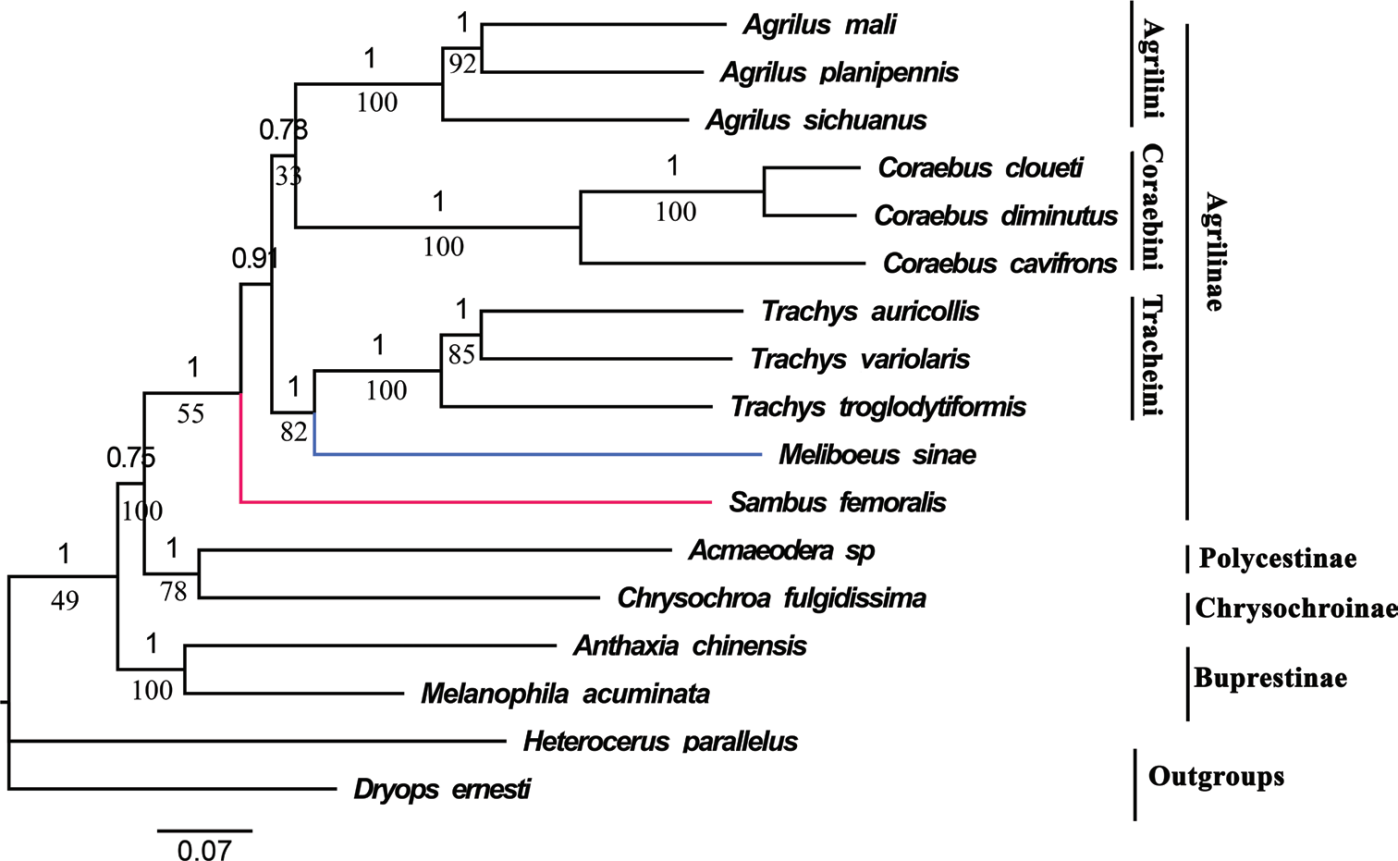
Phylogenetic relationships of 15 selected buprestid species using both BI and ML analyses based on 13 PCGs + 2 rRNAs of mitogenomes. The numbers on the branches show posterior probability (BI tree), whereas the values under branches are bootstrap (ML tree).

Both ML and BI trees using three datasets produced identical topologies (Figs 6–8), (Buprestinae + ((Chrysochroniae + Polycestinae) + Agrilinae)), in terms of subfamily-level relationship. The monophyly of Buprestidae is corroborated again, as all the buprestid species converged together as an independent clade, and two outgroup taxa obviously separated from the buprestid clade. The target species *C.diminutus*, *C.cloueti*, *Meliboeussinae*, *Agrilussichuanus*, and *Sambusfemoralis*, as well as other species of Agrilinae, converged together as an independent clade. And the target species, *M.sinae*, was most closely related to the genus *Trachys* with high value support (Figs 6–8) which is inconsistent with the previous studies (Kubáň et al. 2000; Evans et al. 2015). The relationship of Agrilinae clades obtained from 2 rRNAs and 13 PCGs + 2 rRNAs datasets are identical but with different topology from the 13 PCGs dataset. In the topology generated from the 13 PCGs dataset, *S.femoralis* and *Agrilus* were clustered into a single branch with high support value (Fig. 6, ML: 77, BI: 1) whereas, in the topology generated from the 2 rRNAs and 13 PCGs + 2 rRNAs datasets, *S.femoralis* split from base of the Agrilinae clades (Figs 7, 8). Based on these results the position of the genus *Sambus* in the tribe Agrilini was not suitable and suspect. The different tree topologies suggested that the rRNA genes were extremely valuable for the phylogenetic analysis of Agrilinae. Coraebini is the most diverse tribe in Agrilinae, and 10 subtribes are defined (Kubáň et al. 2000). The genus *Meliboeus* (Meliboeina) and *Coraebus* (Coraebina) in different clades suggested that the tribe Coraebini was polyphyletic, which is consistent with the previous study of Evans et al. (2015). The samples used in this study might be too limited for a comprehensive phylogeny of Buprestidae which still needs a deep study in the future.

## ﻿Conclusions

In this study, five mitogenomes (15,367–16,521 bp) were newly sequenced and annotated, including representatives from the tribes Coraebini and Agrilini in subfamily Agriinae. The mitogenomes of the genera *Sambus* and *Meliboeus* are reported for the first time. These five sequences showed a positive AT skew, and the amino acids Leu, Ile, Phe, Ser2, Gly, Met, and Val were most frequently used. The secondary structures of tRNA-Ser are absent the D-arm, which is similar to other orders of Insecta. The rRNA genes are valuable for phylogenetic analyses of Agrilinae as they could affect the tree topologies. The results show that Coraebini is polyphyletic, and the genus *Sambus* belongs to neither Coraebini nor Agrilini. However, more mitogenome samplings are needed to resolve the phylogeny of the Buprestidae in the future to better understand the phylogenetics of jewel beetles.

## Supplementary materials

Supplementary material 1 Figures S1–S7 Data type Images (pdf file) Explanation note Figure S1. The mitogenome maps of *Agrilussichuanus*, *Coraebuscloueti*, *Coraebusdiminutus*, *Meliboeussinae*, and *Sambusfemoralis*. Figure S2. The secondary cloverleaf structure for the tRNAs of *Agrilussichuanus*. Figure S3. The secondary cloverleaf structure for the tRNAs of *Coraebuscloueti*. Figure S4. The secondary cloverleaf structure for the tRNAs of *Coraebusdiminutus*. Figure S5. The secondary cloverleaf structure for the tRNAs of *Meliboeussinae*. Figure S6. The secondary cloverleaf structure for the tRNAs of *Sambusfemoralis*. Figure S7. Heterogeneous sequence divergence within datasets 13 PCGs and 2 rRNAs of Buprestidae species. https://binary.pensoft.net/file/669401This dataset is made available under the Open Database License (http://opendatacommons.org/licenses/odbl/1.0/). The Open Database License (ODbL) is a license agreement intended to allow users to freely share, modify, and use this Dataset while maintaining this same freedom for others, provided that the original source and author(s) are credited.Zhonghua Wei

## References

[B1] Bellamy CL (1997) Phylogenetic relationships of *Xyroscelis* (Coleoptera: Buprestidae). Invertebrate Systematics 11(4): 569–574. 10.1071/IT94026

[B2] Bellamy CL (2003) An illustrated summary of the higher classification of the superfamily Buprestoidea (Coleoptera). Folia Heyrovskyana (Supplementum 10): 1–197.

[B3] Bellamy CL (2008) A World Catalogue and Bibliography of the Jewel Beetles (Coleoptera: Buprestoidea). Volumes 1–4. Pensoft series faunistica No. 76–79, Sofia/Moscow, [8] + 2684 pp.

[B4] Bellamy CL Volkovitsh M (2016) 18 Buprestoidea Crowson, 1955. In: Beutel RG Leschen RAB (Eds) Handbook of Zoology, Arthropoda: Insecta, Volume 1: Morphology and Systematics (Archostemata, Adephaga, Myxophaga, Polyphaga partim), 2nd edn.Walter de Gruyter, Berlin/Boston, 543–552. 10.1515/9783110373929-021

[B5] Benson G (1999) Tandem repeats finder: A program to analyze DNA sequences. Nucleic Acids Research 27(2): 573–580. 10.1093/nar/27.2.573 9862982 PMC148217

[B6] Bernhard D Fritzsch G Glöckner P Wurst C (2005) Molecular insights into speciation in the *Agrilusviridis*-complex and the genus *Trachys* (Coleoptera: Buprestidae). European Journal of Entomology 102(4): 599–605. 10.14411/eje.2005.083

[B7] Bernt M Donath A Juhling F Externbrink F Florentz C Fritzsch G Stadler PF (2013) MITOS: Improved de novo metazoan mitochondrial genome annotation. Molecular Phylogenetics and Evolution 69(2): 313–319. 10.1016/j.ympev.2012.08.023 22982435

[B8] Cameron SL (2014) Insect mitochondrial genomics: Implications for evolution and phylogeny. Annual Review of Entomology 59(1): 95–117. 10.1146/annurev-ento-011613-162007 24160435

[B9] Cao LM Wang XY (2019a) The complete mitochondrial genome of the jewel beetle *Trachysvariolaris* (Coleoptera: Buprestidae). Mitochondrial DNA, Part B, Resources 4(2): 3042–3043. 10.1080/23802359.2019.1666053 33365847 PMC7706562

[B10] Cao LM Wang XY (2019b) The complete mitochondrial genome of the jewel beetle *Coraebuscavifrons* (Coleoptera: Buprestidae). Mitochondrial DNA, Part B, Resources 4(2): 2407–2408. 10.1080/23802359.2019.1636730 33365565 PMC7687635

[B11] Capella-Gutiérrez S Silla-martínez JM Gabaldón T (2009) TrimAl: A tool for automated alignment trimming in large-scale phylogenetic analyses. Bioinformatics (Oxford, England) 25(15): 1972–1973. 10.1093/bioinformatics/btp348 PMC2712344 19505945

[B12] Chen B Wei ZH Shi AM (2021) The complete mitochondrial genome of the jewel beetle, *Anthaxiachinensis* (Coleoptera: Buprestidae). Mitochondrial DNA, Part B, Resources 6(10): 2962–2963. 10.1080/23802359.2021.1973920 34553059 PMC8451605

[B13] Cline AR Smith TR Miller K Moulton M Whiting M Audisio P (2014) Molecular phylogeny of Nitidulidae: assessment of subfamilial and tribal classification and formalization of the family Cybocephalidae (Coleoptera: Cucujoidea). Systematic Entomology 39(4): 758–772. 10.1111/syen.12084

[B14] Cobos A (1980) Ensayo sobre los géneros de la subfamilia Polycestinae (Coleoptera, Buprestidae) (Parte I). EOS. Revista Española de Entomologia 54: 15–94.

[B15] Cobos A (1986) Fauna Iberica de Coleopteros Buprestidae. Consejo Superior de Invertigaciones Cientificas, Madrid, 364 pp.

[B16] Duan J Quan GX Mittapalli O Cusson M Krell PJ Doucet D (2017) The complete mitogenome of the Emerald Ash Borer (EAB), *Agrilusplanipennis* (Insecta: Coleoptera: Buprestidae). Mitochondrial DNA, Part B, Resources 2(1): 134–135. 10.1080/23802359.2017.1292476 33473743 PMC7800869

[B17] Evans AM Mckenna DD Bellamy CL Farrell BD (2015) Large-scale molecular phylogeny of metallic wood-boring beetles (Coleoptera: Buprestoidea) provides new insights into relationships and reveals multiple evolutionary origins of the larval leaf-mining habit. Systematic Entomology 40(2): 385–400. 10.1111/syen.12108

[B18] Gimmel ML Bocakova M Gunter NL Leschenm RAB (2019) Comprehensive phylogeny of the Cleroidea (Coleoptera: Cucujiformia). Systematic Entomology 44(3): 527–558. 10.1111/syen.12338

[B19] Greiner S Lehwark P Bock R (2019) OrganellarGenomeDRAW (OGDRAW) version 1.3.1: Expanded toolkit for the graphical visualization of organellar genomes. Nucleic Acids Research 47(W1): W59–W64. 10.1093/nar/gkz238 PMC6602502 30949694

[B20] Guindon S Dufayard J Lefort V Anisimova M Hordijk W Gascuel O (2010) New algorithms and methods to estimate maximum-likelihood phylogenies: Assessing the performance of PhyML 3.0. Systematic Biology 59(3): 307–321. 10.1093/sysbio/syq010 20525638

[B21] Hansen JA Moulton JK Klingeman WE Oliver JB Windham MT Trigiano RN Reding ME (2016) Molecular systematics of the *Chrysobothrisfemorata* species group (Coleoptera: Buprestidae). Annals of the Entomological Society of America 108(5): 950–963. 10.1093/aesa/sav080

[B22] Hołyński RB (1988) Remarks on the general classification of Buprestidae Leach as applied to Maoraxiina. Folia Entomologica Hungarica 49(1): 49–54.

[B23] Hołyński RB (1993) A reassessment of the internal classification of the Buprestidae Leach (Coleoptera). Crystal. Series Zoologica (Göd) 1: 1–42.

[B24] Hołyński RB (2009) Taxonomic structure of the subtribe Chrysochroina Cast. with review of the genus *Chrysochroa* Dej. Gondwana, Warszawa, 391 pp.

[B25] Hong MY Jeong HC Kim MJ Jeong HU Lee SH Kim I (2009) Complete mitogenome sequence of the jewel beetle, *Chrysochroafulgidissima* (Coleoptera: Buprestidae). Mitochondrial DNA Mapping, Sequencing, and Analysis 20(2–3): 46–60. 10.1080/19401730802644978 19444700

[B26] Kelnarova I Jendek E Grebennikov VV Bocak L (2019) First molecular phylogeny of *Agrilus* (Coleoptera: Buprestidae), the largest genus on Earth, with DNA barcode database for forestry pest diagnostics. Bulletin of Entomological Research 109(2): 200–211. 10.1017/S0007485318000330 29784069

[B27] Krzywinski J Li C Morris M Conn JE Lima JB Povoa MM Wilkerson RC (2011) Analysis of the evolutionary forces shaping mitochondrial genomes of a Neotropical malaria vector complex. Molecular Phylogenetics and Evolution 58(3): 469–477. 10.1016/j.ympev.2011.01.003 21241811 PMC3046294

[B28] Kubáň V (2016) Tribe Agrilini, genera incertae sedis. In: Löbl I, Löbl D (Eds) Catalogue of Palaearctic Coleoptera. Volume 3, Scarabaeoidea, Scirtoidea, Dascilloidea, Buprestoidea, Byrrhoidea. Revised and updated edition; Leiden, Boston, 549 pp.

[B29] Kubáň V Majer K Kolibáč J (2000) Classification of the tribe Coraebini Bedel, 1921 (Coleoptera, Buprestidae, Agrilinae). Acta Musei Moraviae. Scientiae Biologicae (Brno) 85: 185–287.

[B30] Kubáň V Volkovitsh MG Kalashian MJ Jendek E (2016) Family Buprestidae Leach, 1815. In: Löbl I Löbl D (Eds) Catalogue of Palaearctic Coleoptera.Volume 3, Scarabaeoidea, Scirtoidea, Dascilloidea, Buprestoidea, Byrrhoidea. Revised and Updated Edition. Apollo Books, Stenstrup, 432–574.

[B31] Kück P Meid SA Groß C Wägele JW Misof B (2014) AliGROOVE–visualization of heterogeneous sequence divergence within multiple sequence alignments and detection of inflated branch support. Bioinformatics (Oxford, England) 15: e294. 10.1186/1471-2105-15-294 PMC4167143 25176556

[B32] Kumar S Stecher G Tamura K (2016) MEGA7: Molecular Evolutionary Genetics Analysis version 7.0 for bigger datasets. Molecular Biology and Evolution 33(7): 1870–1874. 10.1093/molbev/msw054 27004904 PMC8210823

[B33] Kundrata R Jäch MA Bocak L (2017) Molecular phylogeny of the Byrrhoidea–Buprestoidea complex (Coleoptera, Elateriformia). Zoologica Scripta 46(2): 150–164. 10.1111/zsc.12196

[B34] Lee MH Lee S Leschen RAB Lee S (2020) Evolution of feeding habits of sap beetles (Coleoptera: Nitidulidae) and placement of Calonecrinae. Systematic Entomology 45(4): 911–923. 10.1111/syen.12441

[B35] Li R Shu XH Li XD Meng L Li BP (2019) Comparative mitogenome analysis of three species and monophyletic inference of Catantopinae (Orthoptera: Acridoidea). Genomics 111(6): 1728–1735. 10.1016/j.ygeno.2018.11.027 30503746

[B36] Librado P Rozas J (2009) DnaSP v5: A software for comprehensive analysis of DNA polymorphism data. Bioinformatics (Oxford, England) 25(11): 1451–1452. 10.1093/bioinformatics/btp187 19346325

[B37] Lowe TM Chan PP (2016) tRNAscan-SE On-line: Integrating search and context for analysis of transfer RNA genes. Nucleic Acids Research 33(W1): W686–W689. 10.1093/nar/gkw413 PMC4987944 27174935

[B38] Park JS Cho Y Kim MJ Nam SH Kim I (2012) Description of complete mitochondrial genome of the black-veined white, *Aporiacrataegi* (Lepidoptera: Papilionoidea), and comparison to papilionoid species. Journal of Asia-Pacific Entomology 15(3): 331–341. 10.1016/j.aspen.2012.01.002

[B39] Pellegrino I Curletti G Liberatore F Cucco M (2017) Cryptic diversity of the jewel beetles *Agrilusviridis* (Coleoptera: Buprestidae) hosted on hazelnut. The European Zoological Journal 84(1): 465–472. 10.1080/24750263.2017.1362050

[B40] Peng XJ Liu J Wang Z Zhan QZ (2021) The complete mitochondrial genome of the pyrophilous jewel beetle *Melanophilaacuminata* (Coleoptera: Buprestidae). Mitochondrial DNA. Part B, Resources 6(3): 1059–1060. 10.1080/23802359.2021.1899079 PMC7995886 33796737

[B41] Pentinsaari M Mutanen M Kaila L (2014) Cryptic diversity and signs of mitochondrial introgression in the *Agrilusviridis*species complex (coleoptera: Buprestidae). European Journal of Entomology 111(4): 475–486. 10.14411/eje.2014.072

[B42] Perna NT Kocher TD (1995) Patterns of nucleotide composition at fourfold degenerate sites of animal mitochondrial genomes. Journal of Molecular Evolution 3(3): 353–358. 10.1007/BF01215182 7563121

[B43] Qin J Zhang YZ Zhou X Kong XB Wei SJ Ward RD Zhang AB (2015) Mitochondrial phylogenomics and genetic relationships of closely related pine moth (Lasiocampidae: *Dendrolimus*) species in China, using whole mitochondrial genomes. Genomics 16(1): e428. 10.1186/s12864-015-1566-5 PMC4455531 26040695

[B44] Robertson JA Ślipiński A Moulton M Shockley FW Gorgi A Lord NP McKenna DD Tomaszewska W Forrester J Miller KB Whiting MF McHugh JV (2015) Phylogeny and classification of Cucujoidea and the recognition of a new superfamily Coccinelloidea (Coleoptera: Cucujiformia). Systematic Entomology 40(4): 745–778. 10.1111/syen.12138

[B45] Ronquist F Teslenko M Der Mark PV Ayres DL Darling AE Hohna S Huelsenbeck JP (2012) MrBayes 3.2: Efficient Bayesian phylogenetic inference and model choice across a large model space. Systematic Biology 61(3): 539–542. 10.1093/sysbio/sys029 22357727 PMC3329765

[B46] Saccone C De Giorgi C Gissi C Pesole G Reyes A (1999) Evolutionary genomics in Metazoa: The mitochondrial DNA as a model system. Gene 238(1): 195–209. 10.1016/S0378-1119(99)00270-X 10570997

[B47] Sheffield NC Song H Cameron SL Whiting MF (2009) Nonstationary evolution and compositional heterogeneity in beetle mitochondrial phylogenomics. Systematic Biology 58(4): 381–394. 10.1093/sysbio/syp037 20525592

[B48] Short AEZ Fikáček M (2013) Molecular phylogeny, evolution, and classification of the Hydrophilidae (Coleoptera). Systematic Entomology 38(4): 723–752. 10.1111/syen.12024

[B49] Song F Li H Liu GH Wang W James P Colwell DD Tran A Gong S Cai WZ Shao R (2019) Mitochondrial genome fragmentation unites the parasitic lice of eutherian mammals. Systematic Biology 68(3): 430–440. 10.1093/sysbio/syy062 30239978 PMC6472445

[B50] Sun HQ Zhao WX Lin RZ Zhou ZF Huai WX Yao YX (2020) The conserved mitochondrial genome of the jewel beetle (Coleoptera: Buprestidae) and its phylogenetic implications for the suborder Polyphaga. Genomics 112(5): 3713–3721. 10.1016/j.ygeno.2020.04.026 32360911

[B51] Thompson JD Higgins DG Gibson TJ (1994) CLUSTAL W: Improving the sensitivity of progressive multiple sequence alignment through sequence weighting, position-specific gap penalties and weight matrix choice. Nucleic Acids Research 22(22): 4673–4680. 10.1093/nar/22.22.4673 7984417 PMC308517

[B52] Tôyama M (1987) The systematic positions of some buprestid genera (Coleoptera, Buprestidae). Elytra 15: 1–11.

[B53] Wang WQ Huang YX Bartlett CR Zhou FM Meng H Qin DZ (2019) Characterization of the complete mitochondrial genomes of two species of the genus *Aphaena* Guérin-Méneville (Hemiptera: Fulgoridae) and its phylogenetic implications. International Journal of Biological Macromolecules 141: 29–40. 10.1016/j.ijbiomac.2019.08.222 31470055

[B54] Wolstenholme DR (1992) Animal mitochondrial DNA: Structure and evolution. International Review of Cytology 141: 173–216. 10.1016/S0074-7696(08)62066-5 1452431

[B55] Xiao JH Jia JG Murphy RW Huang DW (2011) Rapid evolution of the mitochondrial genome in chalcidoid wasps (Hymenoptera: Chalcidoidea) driven by parasitic lifestyles. PLoS ONE 6(11): e26645. 10.1371/journal.pone.0026645 PMC3206819 22073180

[B56] Xiao LF Zhang SD Long CP Guo QY Xu JS Dai XH Wang JG (2019) Complete mitogenome of a leaf-mining buprestid Beetle, *Trachysauricollis*, and its phylogenetic implications. Genes 10(12): e992. 10.3390/genes10120992 PMC6947639 31805706

[B57] Yan L Zhang M Gao Y Pape T Zhang D (2017) First mitogenome for the subfamily Miltogramminae (Diptera: Sarcophagidae) and its phylogenetic implications. European Journal of Entomology 114: 422–429. 10.14411/eje.2017.054

[B58] Yu F Liang AP (2018) The complete mitochondrial genome of *Ugyops* sp. (Hemiptera: Delphacidae). Journal of Insect Science 18(3): e25. 10.1093/jisesa/iey063 PMC6007673 29924333

[B59] Yu P Cheng X Ma Y Yu D Zhang J (2016) The complete mitochondrial genome of *Brachythemiscontaminata* (Odonata: Libellulidae). Mitochondrial DNA A DNA Mapping Sequencing Analysis 27: 2272–2273. 10.3109/19401736.2014.984176 25492539

[B60] Zhang D Gao F Jakovlić I Zou H Zhang J Li WX Wang GT (2020) PhyloSuite: An integrated and scalable desktop platform for streamlined molecular sequence data management and evolutionary phylogenetics studies. Molecular Ecology Resources 20(1): 348–355. 10.1111/1755-0998.13096 31599058

