# Supplementary material for: ﻿The complete mitochondrial genomes of five Agrilinae (Coleoptera, Buprestidae) species and phylogenetic implications
Source: Zookeys. 2022 Apr 6;1092:195–212. doi: 10.3897/zookeys.1092.80993 (PMC9007928; doi:10.3897/zookeys.1092.80993)
Supplement: Supplementary material 1 — Figures S1–S7 [file zookeys-1092-195-s001.pdf]

Supplementary caption:

Figure S1. The mitogenome maps of *Agrilus sichuanus*, *Coraebus cloueti*, *Coraebus diminutus*, *Meliboeus sinae*, and *Sambus femoralis*.

Figure S2. The secondary cloverleaf structure for the tRNAs of *Agrilus sichuanus*.

Figure S3. The secondary cloverleaf structure for the tRNAs of *Coraebus cloueti*.

Figure S4. The secondary cloverleaf structure for the tRNAs of *Coraebus diminutus*.

Figure S5. The secondary cloverleaf structure for the tRNAs of *Meliboeus sinae*.

Figure S6. The secondary cloverleaf structure for the tRNAs of *Sambus femoralis*.

Figure S7. Heterogeneous sequence divergence within datasets 13 PCGs and 2 rRNAs of Buprestidae species.

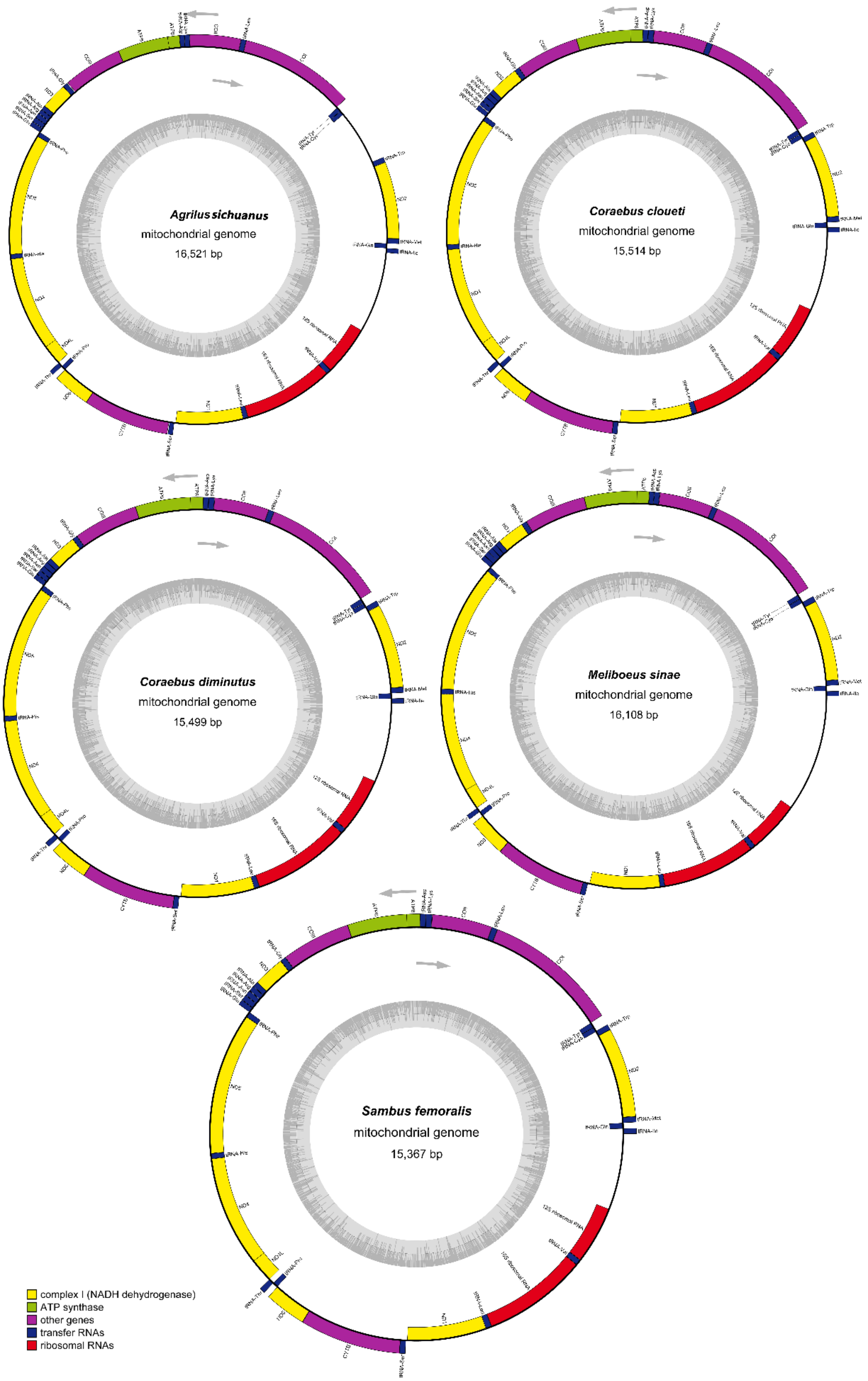

Figure S1. The mitogenome maps of *Agrilus sichuanus*, *Coraebus cloueti*, *Coraebus diminutus*, *Meliboeus sinae*, and *Sambus femoralis*.

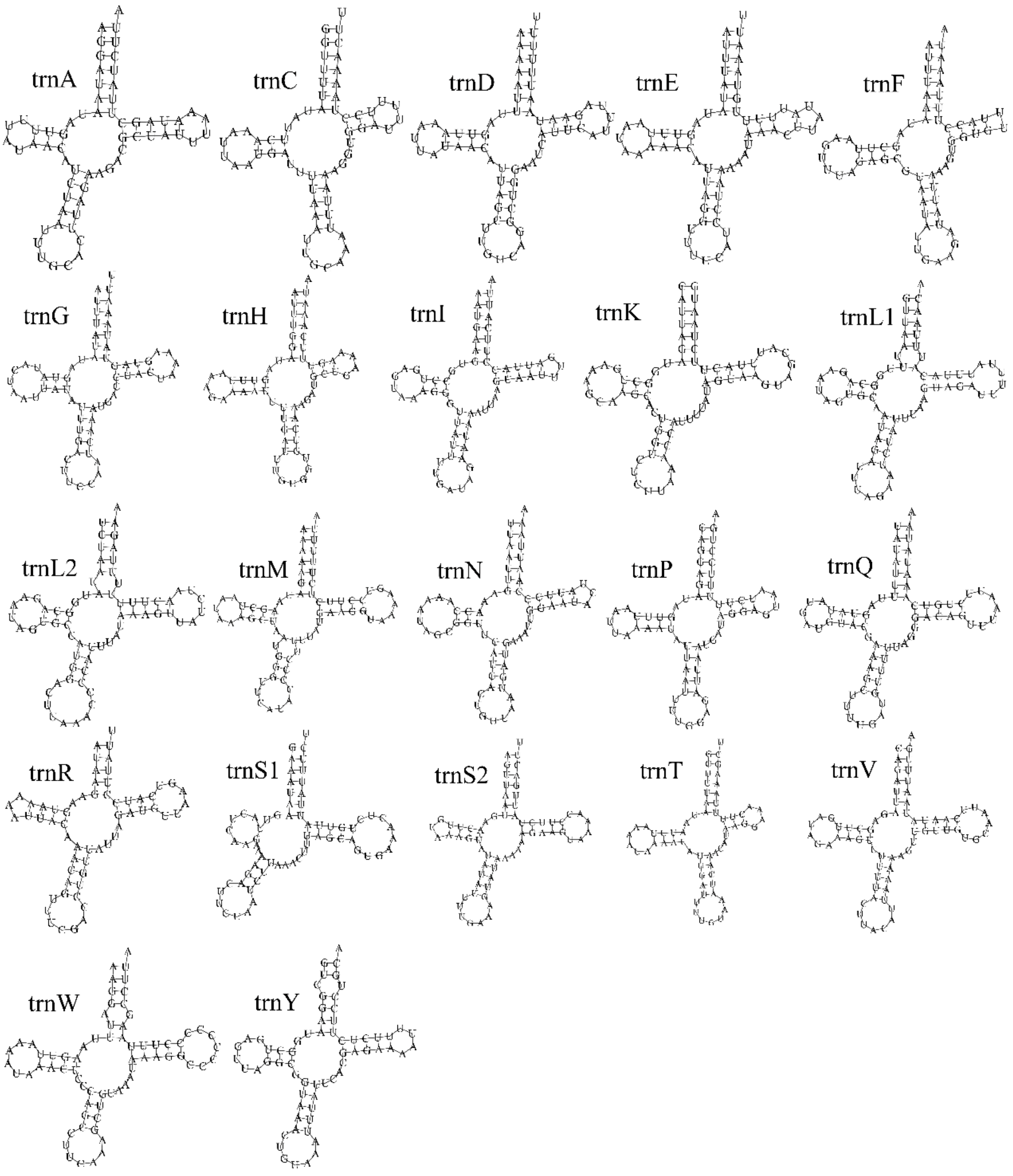

Figure S2. The secondary cloverleaf structure for the tRNAs of *Agrilus sichuanus*.

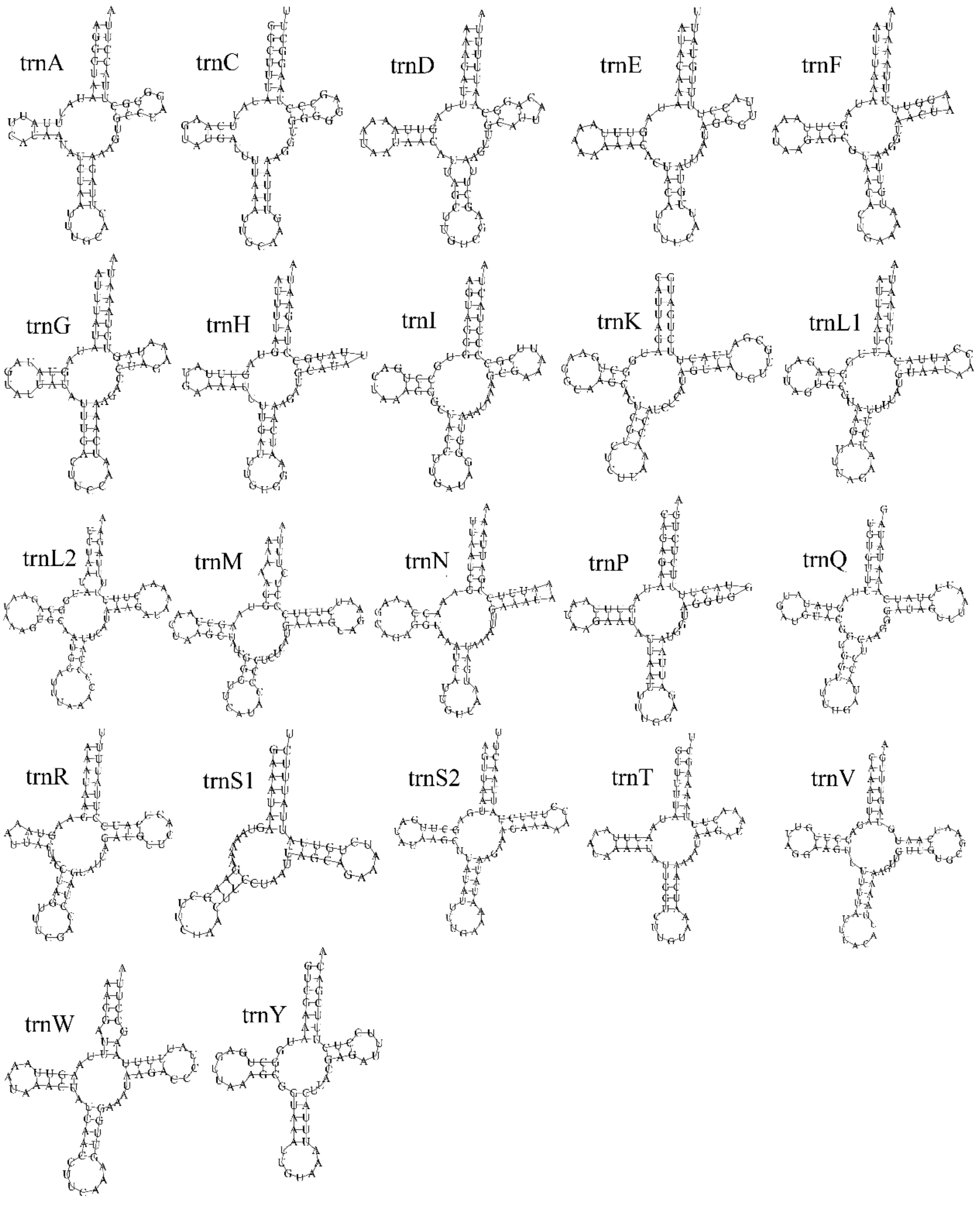

Figure S3. The secondary cloverleaf structure for the tRNAs of *Coraebus cloueti*.

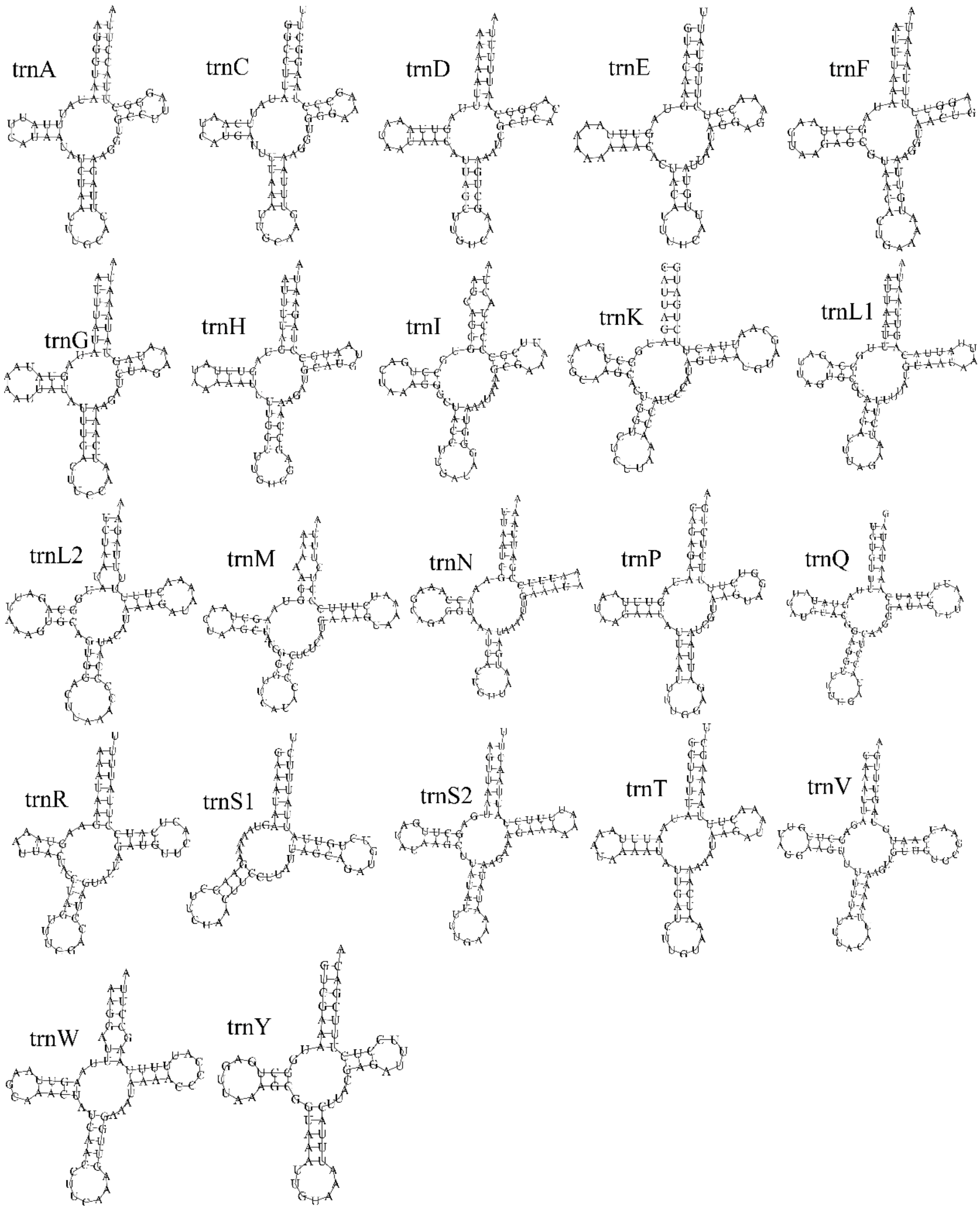

Figure S4. The secondary cloverleaf structure for the tRNAs of *Coraebus diminutus*.

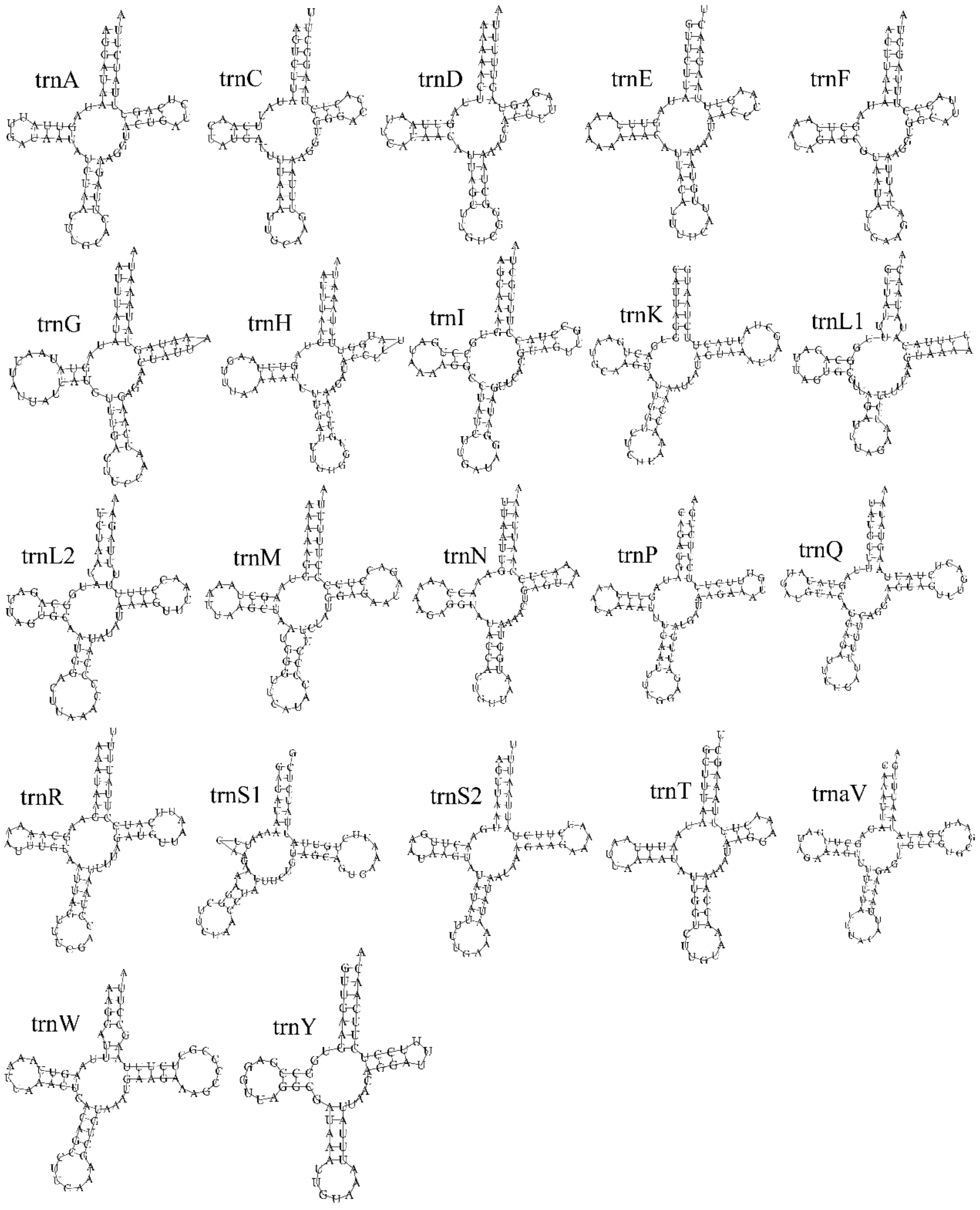

Figure S5. The secondary cloverleaf structure for the tRNAs of *Meliboeus sinae*.

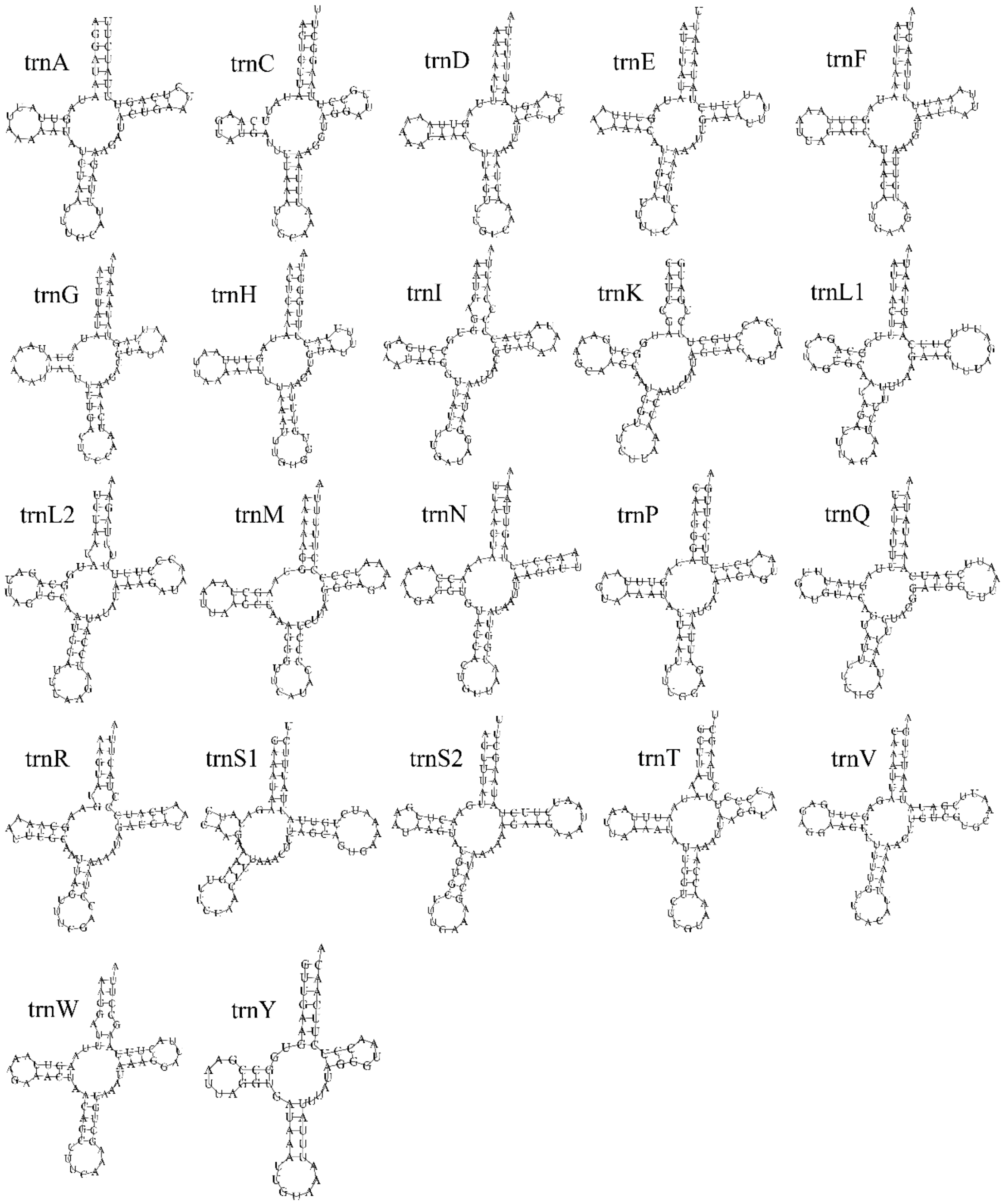

Figure S6. The secondary cloverleaf structure for the tRNAs of *Sambus femoralis*.

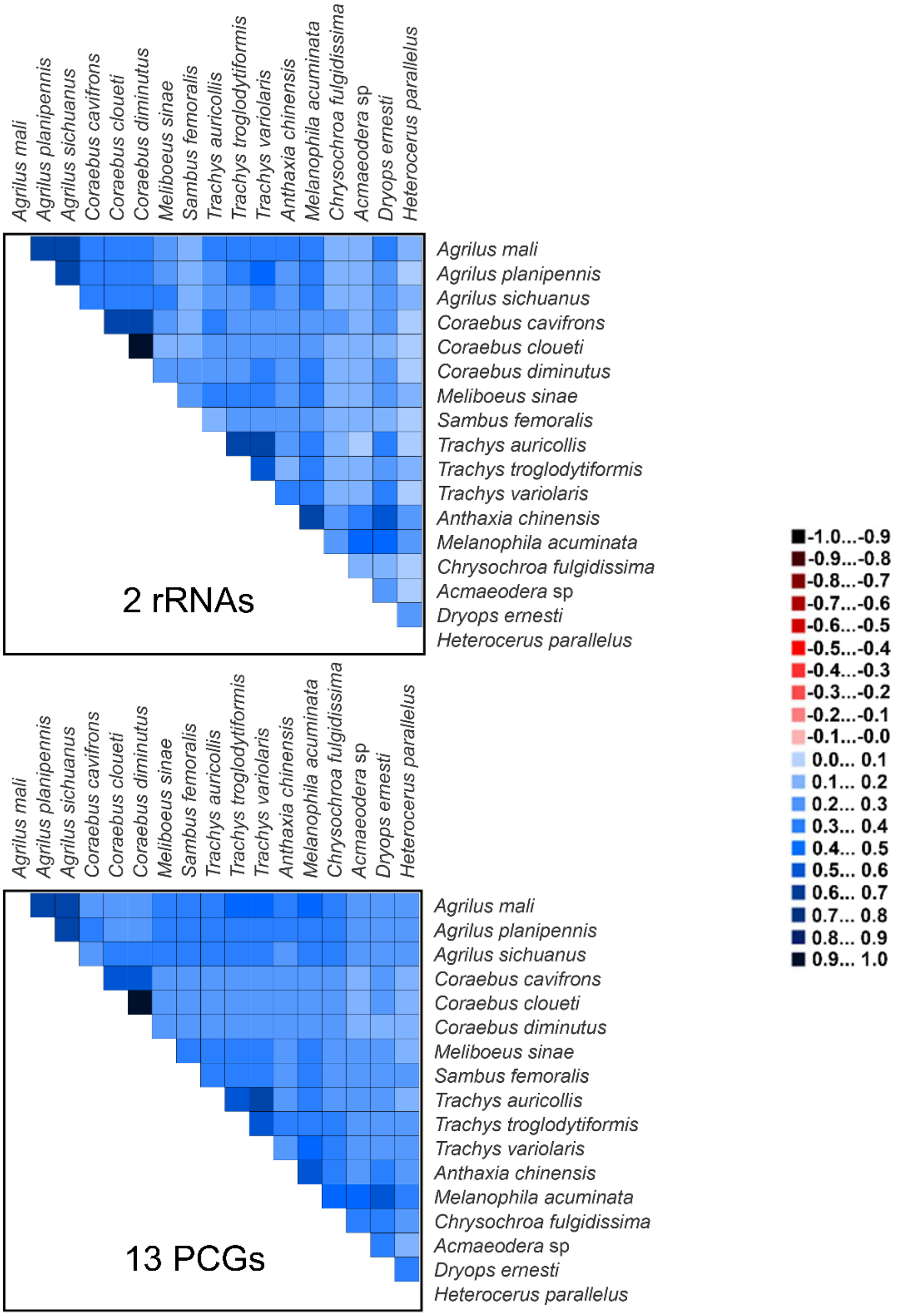

Figure S7. Heterogeneous sequence divergence within datasets 13 PCGs and 2 rRNAs of Buprestidae species
